# On Inflammation of the Iris, and the Influence of the Extract of Belladonna, to Prevent the Consequent Obliteration of the Pupil

**Published:** 1806-07

**Authors:** J. C. Saunders

**Affiliations:** Surgeon


### On Inflammation of the Iris, and the Influence of the Extract of Belladonna, to prevent the consequent Obliteration of the Pupil. *Communicated by* Mr. J. C. Saunders, *Surgeon.*

SCARCELY any disease to which the eye is subject, has a more immediate or rapid tendency to destroy vision, than inflammation of the iris. As soon as this delicate and irritable substance is attacked with inflammation, the brilliancy of its colour fades, it becomes thickened and puckered, the inner margin is turned towards the crystalline lens, and the pupil is exceedingly contracted. The vascularity of the sclerotica is very great, whilst that of the conjunctiva remains much as usual, and it may be easily perceived that the plexus of vessels lies within the latter tunic. The inosculations of those minute vessels are very numerous, and form a species of zone in the junction of the sclerotica and transparent cornea. The vessels disappear at this part as they penetrate the sclerotica, in order to pass to the inflamed iris, and are not continued over the transparent cornea, as in a case of simple ophthalmia.—The irritation on exposure to light is distressing, and the patient is much incommoded by any pressure on the globe of the eye, or by its rapid and sudden motions. Considerable uneasiness is felt over the eye-brow, and acute lancinating pains shoot through the orbit towards the brain. Occasionally, when the inflammation is violent and extends to the other tunics, the eye is totally destroyed by suppuration. But it rarely advances to this extreme. The inflammation generally terminates in the adhesive stage. Lymph is then deposited on the anterior surface of the iris, and between the iris and capsule of the crystalline lens; and often in so large a quantity, as to extend through the pupil and to drop pendulous to the bottom of the anterior chamber. If this process be not interrupted, the pupil is entirely obliterated; or the iris adheres to the capsule of the crystalline lens, leaving only a very minute aperture, which is most commonly occupied by an opake portion of the capsule or organized lymph, and the patient is totally blind. Red vessels appear on the anterior portion of the iris, running in a thin adventitious membrane which the adhesive process causes to be formed. This is the usual catastrophe of an inflamed iris, abandoned to the natural process. It is some consolation to think that this state is not absolutely irremediable. The patient has a chance of

 relief relief by submitting to an operation for cutting an aperture in the iris, and removing the opake capsule and crystalline lens. But it is not my intention to handle this point. My inquiry is entirely directed to the most eligible mode of treating this state of the eye, and preventing the obliteration of the pupil. I am confident I shall be able to shew that inflammation of the iris is manageable, and that, if it should have advanced to the deposition of lymph, and even in a certain degree to the organization of that lymph, the obliteration of the pupil may still be prevented, and a very good degree of vision preserved.

As to the first part of this process, whilst the action of the vessels of the iris is simply increased and no lymph is deposited, we are to be guided by the symptoms abovementioned, viz. the aspect of the iris, the appearance of the vessels on the sclerotica, and the contraction of the pupil, and should immediately have recourse to the most vigorous means of relief. The application of leeches, mild laxatives and a simple regimen, the ordinary practice in a common ophthalmia, will be inadequate. In a healthy person labouring only under this local disease, blood-letting in a degree sufficient to reduce the pulse very considerably, most active cathartics, and deprivation of solid food, will be barely sufficient to stop its progress. For no great degree of action is requisite to complete the mischief; a small quantity of lymph will suffice to unite the iris to the capsule of the lens. In an adult, where there is no contra-indication, 16 to 32 ounces of blood may therefore be taken in the course of twenty-four hours; but the quantity must be regulated by the judgment of the practitioner. I only mean to inculcate the necessity of taking a sufficiency to reduce the pulse and arrest the symptoms, and that if the symptoms should recur as the pulse rises, the bleeding should be repeated. I have generally taken off blood by dividing the temporal artery. Some may be inclined to think that the division of this artery will diminish the quantity of blood in the inflamed parts. But is it not immaterial whence the blood is taken? Is not the benefit which the division of the temporal artery produces simply in proportion to the necessity of reducing the force of the circulation, and therefore in the ratio of the quantity of blood abstracted? Can it be conceived that the division of the artery will be effectual in lessening the quantity of blood, when we consider how many small vessels concur to supply the globe of the eye and parts situated in the orbit? The more minute any set of vessels are, the more frequent frequent their inosculations; and it may fairly be presumed, that if all the vessels were divided which distribute branches to the orbit, and from their situation are divisible, whilst the vis a tergo remained the same, the inflammation would not be reduced. Our chief object is, therefore, to impair the force of the heart, and nothing will more completely accomplish this intention, than the abstraction of blood. Whatever other means medicine furnishes, may be employed with the same view. It may therefore be right, after the exhibition of cathartics, to employ the tartarised antimony in moderated doses, in order to enfeeble the pulse. If vomiting be excited by it, I see no cause of regret, as the straining of the eye in the act of vomiting is more than compensated by the weakness of the pulse, which the state of sickness produces.

General blood-letting, with the means here recommended, will often reduce the inflammation; but, after a degree of general bleeding, which the practitioner may be unwilling to exceed, or in a constitution where it may be injurious, the application of leeches is a powerful auxiliary. The best method is to apply them as close as possible to the eye, and to repeat their application at short intervals, so as to keep up a perpetual oozing of blood from the neighbouring vessels, and to prevent the complete turgescence of those which are inflamed. If the inflammation should stop at this stage, the cure will be completed by covering the eye with a weak solution of ceruss. acetata, and keeping the patient in a darkened room until the iris is restored to the proper exercise of its functions. But this is not the ordinary termination of inflammation of the iris; it generally passes on to the adhesive stage. Lymph is deposited between the iris and capsule, and becoming organized, unites them. Whether the capsule of the lens partakes of the original inflammation, is matter of doubt. I have found in the examination of adhesion, between the iris and capsule of the lens, that the vessels have been derived principally from the iris; and it is a well known fact respecting the formation of adhesions, that when two surfaces are thus joined, most of the vessels proceed from that which is most active, i. e. the surface most inflamed.

It has already been stated, that inflammation of the iris is attended with a remarkable contraction of the pupil; and the lymph which at first simply agglutinates the iris and capsule, ultimately consolidates them, and an immoveable opake substance is interposed between the light and the the immediate organ of vision, the retina. The pupil is seldom *completely* obliterated. An aperture about the size of a pin-hole is left in the iris; but this is rarely beneficial to the patient, as it is occupied by some opake matter. Although in the natural and healthy state of the eye, all the various motions of the iris result from impressions on the retina, to which, as a regulator of the quantity of light, it acts in perfect subserviency, yet the contraction of the pupil, which happens during the inflammation of the iris, must not be regarded as a sympathetic action. On the contrary, its cause is to be sought in the irritation of its muscular fibres, which the inflammation occasions; for however the stimulus of light may be withdrawn from the retina, the contracted pupil remains stationary. The indication in the management of this state of deposited lymph is, to effect as much as possible, the dilatation of the pupil, that when the iris shall be fixed to the capsule of the lens, as it certainly will by the adhesive inflammation, there shall remain a sufficient aperture to transmit light to the bottom of the eye. The larger this aperture the better, as the pupil is generally rendered to a certain degree opake by the lymph which has been deposited on the capsule of the lens. Happily, we are furnished in the extract of belladonna with a perfect specific for this purpose. It is already well known, that the application of this substance to the surface of the conjunctiva excites so strong a contraction in the radiated fibres of the iris, that the pupil is remarkably enlarged, and the whole of the crystalline lens becomes apparent.

We observe certain natural actions in the iris.—We see the pupil diminish by the contraction of its circular fibres; and enlarge by the contraction of the radiated, according as the retina sustains a greater or less degree of light. These are its sympathetic actions with the retina. Again we perceive, that if one eye be shaded, whilst the other is exposed to a strong light, both pupils will be contracted, but not in the same degree as each pupil would be, if both eyes were exposed to the same light. Therefore the iris acts in association with its fellow, and the association is stronger, as the sympathy between the iris and its retina is weakened.

In a case of gutta serena in one eye, the most complete I have ever observed, and which had existed thirty years, the iris annexed to the insensible retina varied precisely as the iris of the sound eye was affected by the changes of light to which it was exposed. The stimulus of the belladonna donna destroys, for a time, both the sympathetic and associated motions of the iris. Under its influence, the radiated fibres are permanently contracted, and the iris does not change its state in obedience to any impulse of light on the retina; much less from association with the other iris. It has, therefore, a specific influence in exciting a strong contraction of the radiated fibres ; and this influence is so great, that in the utmost dilatation of the pupil, which has attended the most perfect insensibility of the retina, I have invariably caused a still greater dilatation by the application of belladonna. Indeed, in a case of trembling cataract, where the lens undulated in the vitreous humour and the iris vibrated with the motions of the eye, as much as a rag floating in a stream would be agitated by the impulse of the current; in this case I say, when from inspection of the iris one would have conceived it to be perfectly inert, I excited a visible action by the belladonna.

Now this substance, if properly applied to the eye during the adhesive process of inflammation, will cause the inner margin of the iris to expand and recede from the axis of the pupil, to overcome the restraint arising from the agglutination of lymph, and to elongate the organized bands which connect the iris and capsule, if they have not been of long duration. Thus the adhesions are drawn out to a degree of tenuity, and consequently transparency, and a considerable quantity of light is admitted. If the effect of the inflammation has been slight, the adhesions will be trivial, and the pupil only slightly irregular. The iris will retain a certain power of action, and vision will be very little injured. In general the pupil is mis-shapen, and the iris perfectly fixed; but if the aperture be of sufficient size, and the capsule not rendered too opake, the patient will enjoy a very useful degree of sight.—The reader will observe, that in this communication I have been speaking of inflammation of the iris, as of a disease which I have often seen uncombined with any superficial ophthalmia. It must, however, be granted, that generally an inflammation of the conjunctiva, in a greater or less degree, is associated with it; but unless there be deep ulcerations, or sloughing of the cornea, the treatment of the case will not materially vary. But this state of the iris sometimes arises from syphilis. Then the general plan of treatment here proposed must be changed for the specific remedy, and mercury must be vigorously exhibited, if it be proposed to obviate the effect of inflammation, which is the same whether the inflaminflammation be general or specific; the use of the belladonna is therefore equally advantageous.

### CASE I.

James Bradshaw, a young man, robust, and of a florid complexion, applied at the Dispensary, March 27th, 1805, being afflicted with a violent ophthalmia that made him blind in the right eye. There were evident marks of inflammation of the iris, but this was complicated with a considerable superficial ophthalmia and diffusion of lymph in the transparent cornea. He was treated much according to the plan inculcated in this Essay for the space of a fortnight, but without any success. Disappointed in the employment of means, which I thought must have proved effectual in suppressing pure inflammation, I was induced to investigate the case with the most particular attention. I found on examination a painful and contracted state of the elbow, but no enlargement of the bones or thickening of the ligaments. This symptom, in conjunction with the state of the eye, determined me to treat the case as syphilis. In the space of ten days the inflammation had decreased, the transparent cornea was clear, and the state of the iris perceptible. The pupil was very minute, and evidently opake; and the patient, notwithstanding the restoration of the cornea to its original transparency, could barely perceive the largest objects. The mercury was continued, and the inflammation had subsided, still the sight improved very slowly, in consequence of the contraction of the pupil. I now applied the extract of belladonna three times in the day. In the course of twenty-four hours the pupil was drawn into a most irrègular figure. Two bands, attached to the inner margin of the iris, and joining each other like the letter T, cut the pupil as it were into three. These bands were gradually elongated, and became extremely minute. The belladonna was continued to the latter end of June. When the pupil had nearly recovered its circular form, and his vision being extremely good, scarcely, if at all, inferior to that of the other eye, he ceased to attend any longer at the Dispensary.

### CASE II.

J. Richardson applied at the Dispensary, Nov. 29th, 1805, on account of acute inflammation of the iris; the pupil being much contracted and opake from the deposition of lymph. This eye was nearly blind on his admission: He

He lost a pound of blood by opening the temporal artery, and was treated in other respects on the plan previously laid down. Dec. 6th, the inflammation had subsided, but the pupil still remained contracted and opake, and vision very imperfect. The extract of belladonna was now applied three times a day; and at the same time, proper means were taken to expedite the absorption of the lymph. Jan. 20th, he left the Dispensary, being restored to perfect vision. The iris enjoyed a certain degree of action, but the pupil could not dilate to an equal degree with that of the other eye, the iris being restrained by its attachment to the capsule of the lens.

### CASE III.

Jan. 23d, 1806, Mary Skinner, a woman of a plethoric habit, applied at the Dispensary, labouring under acute inflammation of the eye. The pupil was contracted, and rendered opake by a deposition of lymph. She was blind on her admission. The temporal artery was divided, and she was otherwise treated on an active antiphlogistic plan. On the 31st, inflammation had subsided, the pupil a little clearer, and she could distinguish large objects. Feb. 5th, pupil still contracted as at first. I now applied the extract of belladonna: in the course of two days the pupil was considerably enlarged and oval; and by the exhibition of proper remedies, the lymph occupying the aperture was nearly absorbed. The iris, however, remains fixed by adhesions, and the pupil does not vary. Her sight is very good.

### CASE IV.

Ann Row, Feb. 14th, 1806, applied at the Dispensary, after an ophthalmia of the left eye, which had spontaneously subsided. She remained blind. On examination of her eye, the pupil was very small, oval, snd occupied by lymph. No symptom of inflammation was now present. She took calom. gr. ij. every night, and every other morning a brisk cathartic. The extract of belladonna was applied three times a day. The pupil enlarged under its application to a very proper size, but was oval, considerably opake, and perfectly fixed by adhesion of the iris to the capsule. Enough was clear to admit of her seeing objects of moderate size. She could distinguish well enough to tell the time by a watch within a second or two. The defect arose, not from the contraction of the pupil (that was sufficiently opened opened by the belladonna,) but from the remaining opacity of the capsule. She left the Dispensary, April 10th, as I could not render any more service by the continuance of this plan.

April 23d, 1806, Margaret Onbird was recommended to my care by Mr. R. Pugh, jun. of Gracechurch-street, being blind in one eye In this instance an ophthalmia, which had not been very severe, had terminated spontaneously some time before her application. The pupil was contracted, irregular, and opake. She was treated precisely as the case recited above. In the course of a fortnight the pupil was much enlarged, but nothing could be more irregular, in consequence of the adhesions being elongated by the retraction of the iris. She has continued the plan to the present time. The pupil is less irregular, having assumed an oval form, and is tolerably clear. She can now read a moderate print with this eye; but on her application could not perceive a single letter of the largest dimensions. The pupil, although large enough, is perfectly fixed, as in the former instance.

*June*, 16, 1806.

